# Supplementary material for: Sugar Import Suppresses Klebsiella pneumoniae Mucoidy in cAMP-CRP-dependent Manner
Source: bioRxiv. 2025 Nov 4:2025.11.04.686645. Preprint. [Version 1] doi: 10.1101/2025.11.04.686645 (PMC12637589; doi:10.1101/2025.11.04.686645)
Supplement: 1 [file NIHPP2025.11.04.686645v1-supplement-1.pdf]

bioRxiv preprint doi: https://doi.org/10.1101/2025.11.04.686645; this version posted November 4, 2025. The copyright holder for this preprint (which was not certified by peer review) is the author/funder, who has granted bioRxiv a license to display the preprint in perpetuity. It is made available under aCC-BY 4.0 International license.

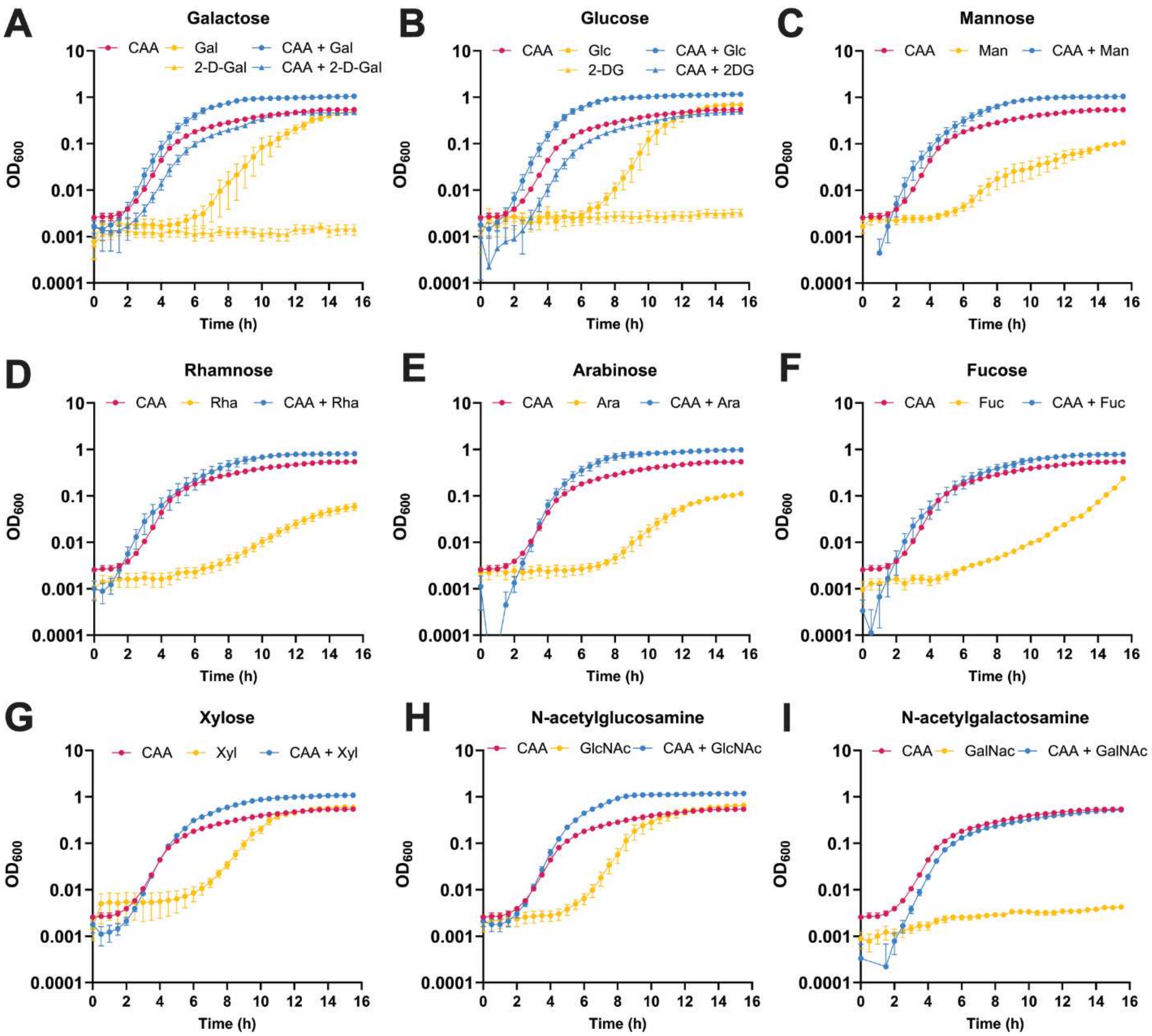

Supplementary Figure 1. ***K. pneumoniae*** **grows on most individual sugars, which increases when combined with casamino acids.** KPPR1 was cultured in M9+CAA (1%), M9+sugar (80 mM) and M9+CAA+sugar. After inoculating KPPR1 in each growth medium, OD_{600} was measured every 30 minutes for 16 hours at 37˚C. Data presented are the mean, and error bars represent the standard error of the mean. Experiments were performed 3 independent times, in triplicate.

bioRxiv preprint doi: https://doi.org/10.1101/2025.11.04.686645; this version posted November 4, 2025. The copyright holder for this preprint (which was not certified by peer review) is the author/funder, who has granted bioRxiv a license to display the preprint in perpetuity. It is made available under aCC-BY 4.0 International license.

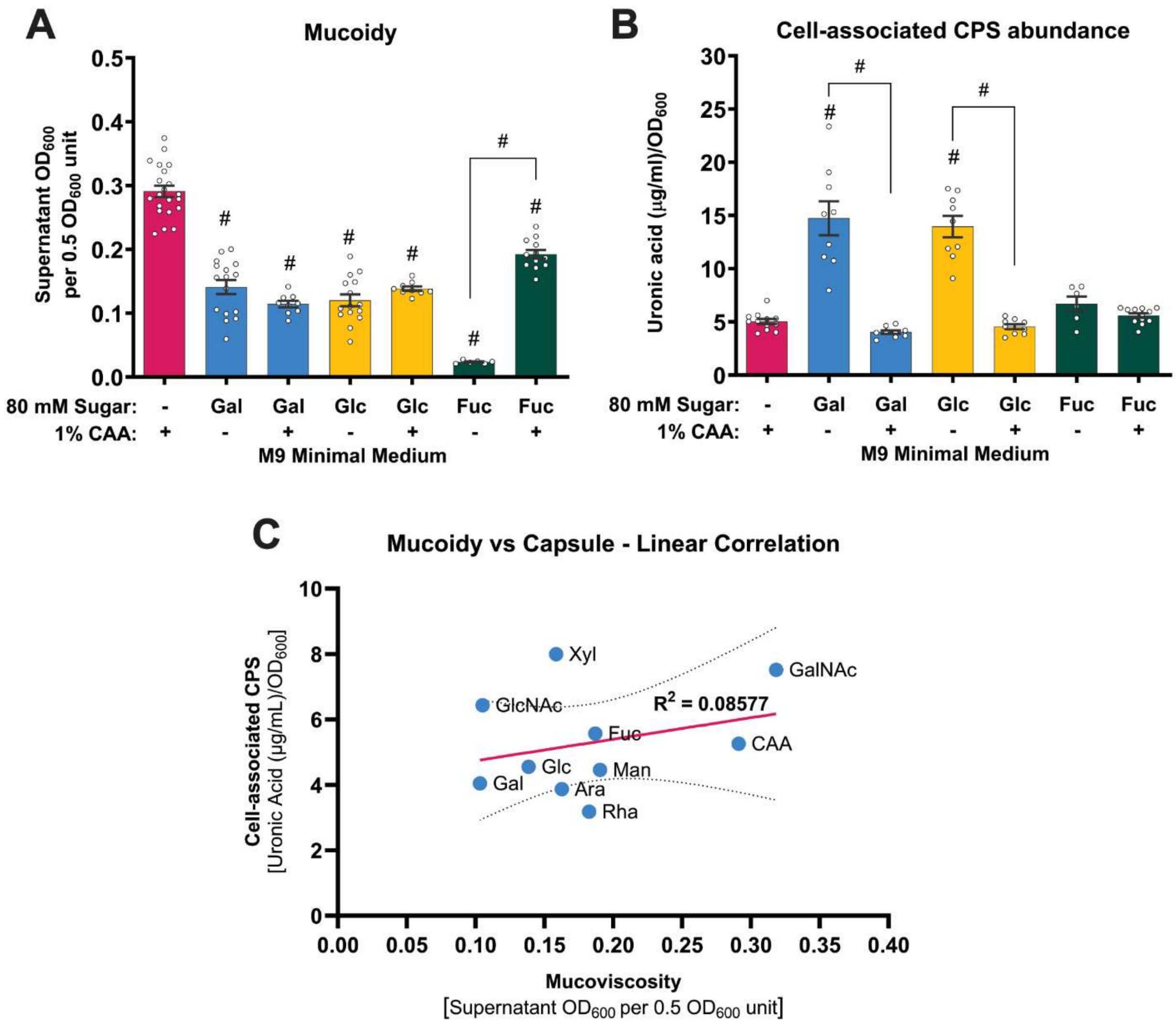

Supplementary Figure 2. Sugars suppress *K. pneumoniae* mucoidy independent of CPS abundance. (A and **B)** KPPR1 was cultured in M9+CAA, M9+sugar and M9+CAA+sugar. **(A)** Mucoidy was determined by quantifying the supernatant OD_{600} after sedimenting 0.5 OD_{600} unit of culture at 1,000 x *g* for 5 mins and **(B)** uronic acid abundance was quantified for total CPS and supernatant CPS and normalized to OD_{600}. Cell-associated CPS abundance was calculated by subtracting supernatant CPS from the total CPS content. **(C)** Mucoidy and cell-associated CPS abundance presented in **Fig. 1A** and **1C** were analyzed for correlation using simple linear regression. **(A-B)** Data presented are the mean, and error bars represent the standard error of the mean. Statistical significance was determined using one-way ANOVA with Šídák correction. Statistical significance was calculated by comparing sugar-supplemented condition to M9+CAA or between adjacent pairs of bars. # $p \leq 0.0001$. Experiments were performed ≥3 independent times, in triplicate.

bioRxiv preprint doi: https://doi.org/10.1101/2025.11.04.686645; this version posted November 4, 2025. The copyright holder for this preprint (which was not certified by peer review) is the author/funder, who has granted bioRxiv a license to display the preprint in perpetuity. It is made available under aCC-BY 4.0 International license.

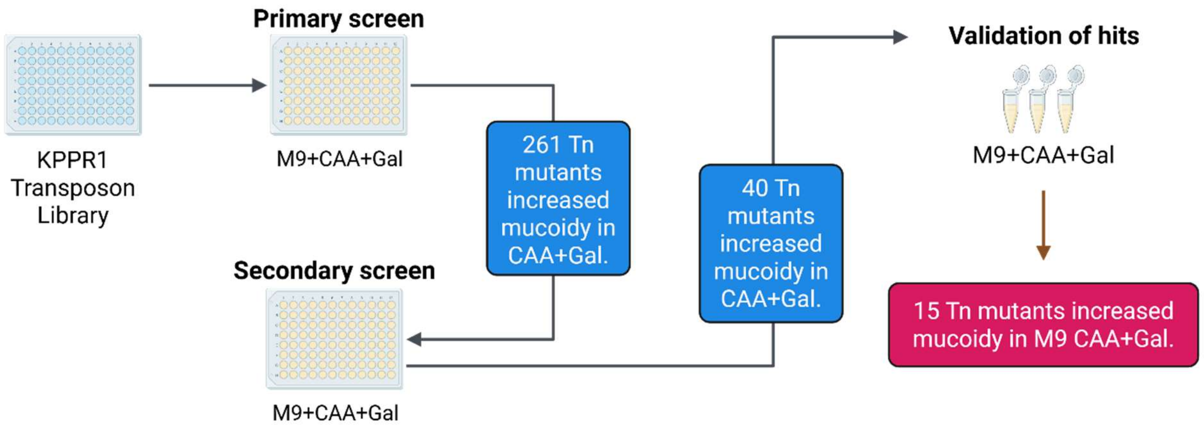

Supplementary Figure 3. Graphical illustration of transposon screening workflow to identify mutants with increased mucoidy in galactose-supplemented medium. Primary and secondary screen were performed in M9+CAA+Gal using a sedimentation assay adapted to a 96-well plate format for high-throughput screening. Final validation of hits was performed by a tube-based sedimentation assay. Data are presented in **Figure 5A**. Mutants with mucoidy higher than two times the standard deviation of average mucoidy of a plate were identified as primary hits. Mutants with mucoidy significantly higher than WT in M9+CAA+Gal were confirmed as validated hits (*n* = *15*).

bioRxiv preprint doi: https://doi.org/10.1101/2025.11.04.686645; this version posted November 4, 2025. The copyright holder for this preprint (which was not certified by peer review) is the author/funder, who has granted bioRxiv a license to display the preprint in perpetuity. It is made available under aCC-BY 4.0 International license.

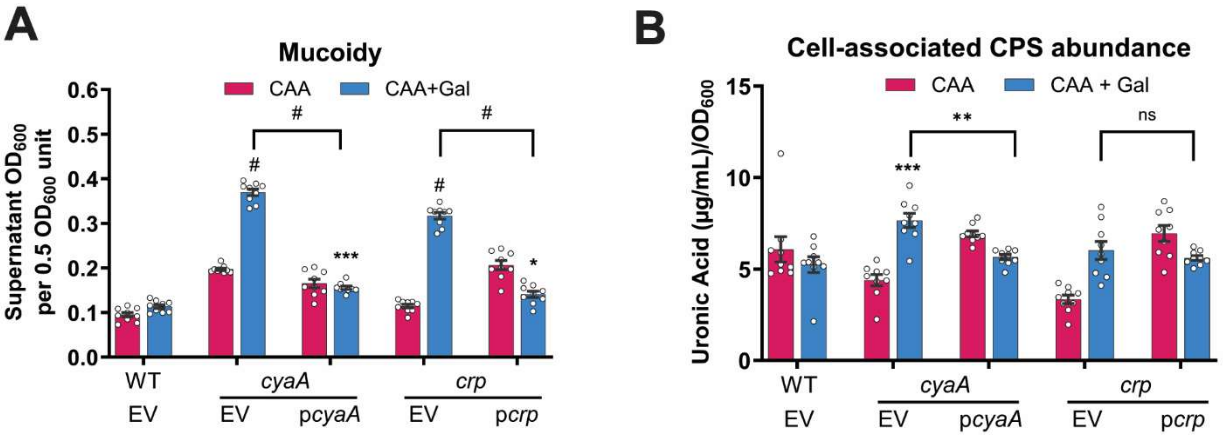

Supplementary Figure 4. Complementation of *cyaA* and *crp* deletion strains. KPPR1 WT with empty vector (WT+EV), and *cyaA* and *crp* mutants with EV (*cyaA*+EV and *crp*+EV) or their respective complementation vectors (*cyaA*+p*cyaA* and *crp*+p*crp*) were cultured in M9+CAA±Gal. **(A)** Mucoidy was determined by quantifying the supernatant OD_{600} after centrifugation at 1,000 x *g* for 5 mins. **(B)** Cell-associated CPS was extracted and measured for uronic acid content. Data presented are the mean, and the error bars represent the standard error of the mean. Statistical significance was determined using two-way ANOVA with Šídák correction, by either comparing mutant-based strains in M9+CAA+Gal to WT in M9+CAA+Gal (*p*-value above each bar) or EV to the respective complementation vector (*p*-values above each connected line). * $p \leq 0.05$; ** ≤ 0.01; *** $p \leq 0.001$; # $p \leq 0.0001$. All experiments were performed ≥3 independent times, in triplicate.

bioRxiv preprint doi: https://doi.org/10.1101/2025.11.04.686645; this version posted November 4, 2025. The copyright holder for this preprint (which was not certified by peer review) is the author/funder, who has granted bioRxiv a license to display the preprint in perpetuity. It is made available under aCC-BY 4.0 International license.

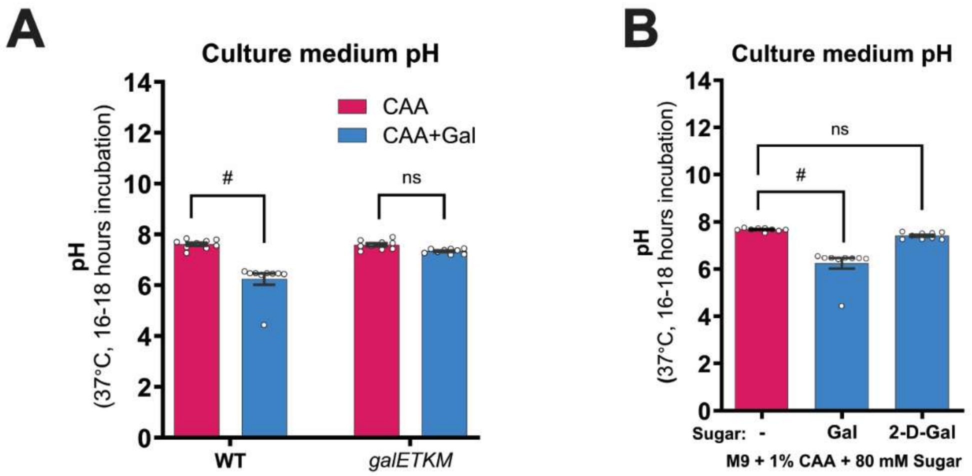

Supplementary Figure 5. KPPR1 *galETKM* mutant and 2-deoxy-D-galactose do not decrease growth medium pH. (A) KPPR1 WT and *galETKM* mutant were cultured in M9+CAA±Gal. **(B)** KPPR1 WT was cultured in M9+CAA supplemented with Gal or the non-metabolizable analog, 2-D-Gal. For both **(A** and **B),** bacterial strains were cultured in their respective growth media at 37˚C for 16-18 hours, then the culture medium pH was measured using a pH meter. Data presented are the mean, and error bars represent the standard error of the mean. Statistical significance was determined using **(A)** two-way ANOVA with Šídák correction or **(B)** one-way ANOVA with Dunnett's post-hoc test. Statistical significance was calculated by comparing sugar-supplemented condition to M9+CAA. # $p \leq 0.0001$; ns = non-significant. Experiments were performed 3 independent times, in triplicate.
